# Differential expression dynamics of Growth differentiation factor9 (*GDF9*) and Bone morphogenetic factor15 (*BMP15*) mRNA transcripts during *in vitro* maturation of buffalo (*Bubalus bubalis*) cumulus–oocyte complexes

**DOI:** 10.1186/2193-1801-2-206

**Published:** 2013-05-06

**Authors:** Muralidharan Kathirvel, Eswari Soundian, Vijayarani Kumanan

**Affiliations:** Department of Veterinary Physiology, Laboratory of Reproductive Physiology, Madras Veterinary College, Chennai, 600 007 TANUVAS, Tamil Nadu India; Department of Veterinary Physiology and Biochemistry, Veterinary College and Research Institute, Tirunelveli, 627 001 TANUVAS, Tamil Nadu India; Department of Animal Biotechnology, Madras Veterinary College, Chennai, 600 007 TANUVAS, Tamil Nadu India

**Keywords:** *BMP15*, COC, Expression, *GDF9*, IVM

## Abstract

The present study has evaluated the association of growth differentiation factor9 (*GDF9*) and bone morphogenetic protein15 (*BMP15*) mRNA expression in cumulus-oocyte complexes (COCs) of buffalo ovary during *in vitro* maturation (IVM). *GDF9* and *BMP15* are expressed specifically in mammalian oocytes and also participate in cumulus-oocyte crosstalk. Quantitative real-time polymerase chain reaction (qRT-PCR) technique was applied to investigate the relative abundance (RA) of *GDF9* and *BMP15* mRNA transcripts throughout the IVM process. Relative mRNA expression pattern of these specific genes were assessed in oocytes and cumulus cells at 0, 6, 12 and 24 h of *in vitro* culture. Our results revealed that RA of *GDF9* during different hours of IVM showed significant reduction between 0 h and 24 h of maturation in oocytes and *BMP15* transcript increased significantly (P<0.05) between 6 h and 12 h and decreased again between 12 h and 24. In cumulus cells, *GDF9* remained stable during IVM upto 12 h of maturation and decreased significantly between 12 h and 24 h of maturation. Conversely, significant reduction of *BMP15* was observed between 0 h and 6 h, stayed stable upto 12 h and became undetectable at 24 h of maturation. In conclusion, these two genes were differentially expressed during the period of oocyte maturation process and notably, *BMP15* expression pattern is associated specifically with the period of cumulus cell expansion.

## Introduction

Growth factors synthesized from mammalian oocytes popularly known as oocyte secreted factors (OSFs) play numerous role in ovarian folliculogenesis. In particular, a growing body of evidence in recent years indicated that two famous members of Transforming growth factor-β superfamily, *GDF9* and *BMP15* have been involved in the control of ovulation rate in mammals (.Moore *et al.*^2004^). They act synergistically to affect development of the cumulus–oocyte complexes in mice (Yan *et al.*^2001^). *GDF9* plays critical roles in granulosa and theca cells growth, as well as in differentiation and maturation of the oocyte (Hreinsson *et al.*^2002^). Further, the level of *GDF9* in human follicular fluid has been found significantly correlated with the nuclear maturation of the oocytes and embryo quality (Gode *et al.*^2011^). Moreover, *BMP15* has also been thought to be involved in oocyte maturation and follicular development alone or along with the related protein, *GDF9*. Both the proteins are progressively expressed by the oocytes of growing follicles throughout folliculogenesis (Dube *et al.*^1998^; Laitinen *et al.* 1998). This stage specific expression through intra-follicular cascade at the correct time is vital for follicular growth in order to reach the ovulatory phase (Campbell 2009). In this regard, the expression of *GDF9* and *BMP15* has been studied extensively in follicular compartment cells in the ovaries of various species, while poorly explored in the COCs within culture systems.

The domestic Asian water buffalo (*Bubalus bubalis*) is a multipurpose livestock animal. It is of particular important production animal due to the source of high quality animal protein in developing countries (Zicarelli 1994). IVM is an essential technology under assisted reproductive technologies (ARTs), which enables oocytes to achieve maturation and acquire the competence for subsequent embryonic division leading to blastocyst formation (Lonergan *et al.*^2003^; Somfai *et al.*^2011^). This technique is valuable not only because they allow the production of large numbers of oocytes, but also because they provide a valuable *in vitro* model to study the gene expression at different maturational stages of oocytes. During the maturation process, oocytes accumulate large amount of maternal mRNAs to dictate the developmental competence of oocytes (Watson 2007). Quantification of RNA developmentally important oocyte secreted factors in oocytes during maturation might be valuable when setting up IVM medium that lack the normal hormonal interplay found *in vivo*. Considering the importance of buffalo and the necessity of designing potent ART protocols for this species, the current study aimed for analysing expression pattern of two oogenesis specific genes during maturation. For this purpose, we used quantitative real-time PCR (qRT-PCR) technique for mRNA expression of GDF9 and BMP15 genes. Accordingly, the current work will provide important leads for understanding the mRNA transcriptional level of *GDF9* and *BMP15* genes in oocytes during IVM in case of buffalo.

## Materials and methods

All chemicals and culture media were obtained from Sigma Chemical Co. (USA) unless otherwise stated. Disposable plastic wares used were purchased from Nunc, Denmark.

### Collection of cumulus oocyte complexes and IVM

Buffalo ovaries were collected from an abattoir and transported in thermos containing pre-warmed (37°C) normal saline to the laboratory within 2-3 h. In the laboratory, ovaries were washed five times in modified Dulbecco’s phosphate buffered saline (mDPBS) containing gentamicin. Oocytes were aspirated with a 10 cc syringe using 20 gauge needle into 50 ml conical tubes from follicles (>3 mm in diameter) of buffalo ovaries. The aspirated COCs were graded based on morphological appearance of the cumulus cell investments and homogeneity of ooplasm under stereo zoom microscope as described by Nandi *et al.* (1998) and the different grades are, Grade A (Excellent): COCs with more than 4 layers of compact cumulus cells with evenly granular homogenous ooplasm; Grade B (Good): COCs with 2–4 layers of compact cumulus cells with evenly granular homogenous ooplasm; Grade C (Poor): COCs with one layer of cumulus cells with irregular dark ooplasm; Grade D (Poor): Naked oocytes with irregular dark ooplasm. The aspirated oocytes of grade A and B alone were chosen for *in vitro* maturation studies. Average of 20–30 oocytes were cultured in maturation media and each experiment was replicated on eight separate occasions. From 851 ovaries, total of 749 oocytes were used for this study. Maturation medium consisted of TCM-199 supplemented with 10% FBS, 10 IU/ml LH, 0.5 μg/ml FSH, 1 μg/ml estradiol-17β, 100 IU/ml penicillin and 100 μg/ml streptomycin. The oocytes were subjected to mature in the maturation medium in 35×10 mm petridishes for 24 h in a CO_2_ incubator maintained at 39°C, 5% CO_2_ with maximum relative humidity.

### Assessment of oocyte maturation

The maturation of oocytes was evaluated under stereomicroscope based on the degree of cumulus expansion (Kobayashi *et al.*^1994^) and further evaluated by identifying the first polar body in the perivitelline space when the oocytes were denuded after maturation. For this purpose, a proportion of COCs were recovered from the culture and transferred to 1.5 ml tube containing 400 μl of 0.1% trypsin-EDTA solution. Then vortex-agitated for 1 min to remove cumulus cells and thus denuded oocytes were visualised for extrusion of polar body.

### Collection of samples for expression analysis

Immature oocytes (0 h) were treated with 0.1 per cent trypsin-EDTA for 2–3 min and mechanically denuded of surrounding cumulus cells by repeated pipetting. The cumulus-free oocytes were observed under stereo zoom microscope to ensure that they were free of cumulus cells. The oocytes were washed twice in PBS and transferred to a fresh 1.5 ml micro centrifuge tube with minimum volume of PBS and then frozen at −80°C. After the removal of cumulus free oocytes, the remaining cumulus cells in PBS were transferred to micro centrifuge tube and centrifuged at 300 g for 2 min according to Caixeta *et al.* (2009). The supernatant was removed and the cell pellet was resuspended with minimum volume of PBS and stored at −80°C for RNA isolation. During different hours of IVM (6, 12 and 24 h), the cumulus denuded from matured oocytes were collected as mentioned above and the oocytes with evenly granular ooplasm after cumulus removal with a visible polar body in the peri-vitelline space were also stored at −80°C for RNA isolation.

### RNA isolation and cDNA preparation

Total RNA was extracted from pools of immature (IM 75), *in vitro* matured oocytes (MO 50), cumulus cells (cells removed from 50–75 COCs) using RNeasy Plus Mini Kit (Qiagen, USA) and eluted in 30 μl RNase free-water. The purity and concentration of isolated RNA was determined by ND1000 spectrophotometer (Nanodrop technologies, USA) and purity was assessed using the A260/A280 nm ratio with expected values between 1.8 and 2.0. The cDNA synthesis was carried out with 6 μl of RNA using Superscript III first-strand synthesis system (Invitrogen, USA) and random hexamer primer in a final reaction volume of 25 μl. The cDNA synthesis reactions were carried at 25°C for 10 min for annealing, 50°C for 60 min for extension, followed by heat inactivation of the enzyme at 85°C for 5 min. Reactions without Reverse transcriptase served as controls. After termination of cDNA synthesis, each RT reactions were diluted with nuclease-free water to a final volume of 50 μl and stored at −80°C till further use.

### Primer designing and validation

Oligonucleotide primers for qRT-PCR analysis were designed from *Bubalus bubalis* complete mRNA sequence of GDF-9 (GenBank: FJ529501.2), BMP-15 (GenBank: EF375880.1) and GAPDH (GenBank: GU324291.1). Oligo Analyzer (1.1.2 Version) software was used to determine the Tm, GC per cent, primer loops, primer dimers and primer-primer compatibility. The FastPCR Professional (6.2.45 beta 4 version) has been used for Real time PCR primer designing and the oligonucleotide primers were synthesized from Sigma-Aldrich (USA). The designed primers were optimized prior to quantification experiments using polymerase chain reaction. For these genes, the expected sizes of the products were confirmed by gel electrophoresis on a 2% agarose gel. The primer sequences, expected fragment size of amplified products and genbank accession numbers are shown in Table 1.

**Table 1:** **Primers used for qRT-PCR analysis**

Gene	Primer sequence (5′→ 3′)	Size (bp)	GenBank accession No.
**GDF-9**	F: TGCTCAGGCTTTTCACAGGTGGCA	131	FJ529501.2
	R: GACGGGACAATCTTACACCCTCAG		
**BMP-15**	F: TGGTGAAGCAGGCGTCAGAG	167	EF375880.1
	R: CGCCAGTAGAAGCAGGGGTGAT		
**GAPDH**	F: CCTGGAGAAACCTGCCAAGTA	139	GU324291.1
	R: GGTAGAAGAGTGAGTGTCGCT		

### Real-time polymerase chain reaction

Real-time PCR was performed to quantify the mRNA transcripts of GDF-9, BMP-15 in oocytes and cumulus cells using SYBR Green JumpStart Taq ReadyMix kit from Sigma-Aldrich, USA. Each cDNA sample was analysed in duplicate using real-time thermal cycler (Applied Biosystems, USA, ABI 7500). A non template control (NTC) was prepared using DEPC water without cDNA. Reactions were performed in 20 μl volumes and loaded in the real-time qPCR 96-well plate (10 μl/well) and the plate was centrifuged in cooling (4°C) plate at 560 rpm for 2 min to remove the air bubbles and kept for reaction in real time thermal cycler.

The PCR cyclic conditions were 95°C for 10 min followed by 40 cycles of denaturing at 95°C for 15 seconds and then annealing (60°C for GDF-9, 56°C for BMP-15, 58°C for GAPDH) for 1 min. The standard curve was obtained for GAPDH, *GDF9* and *BMP15* genes using 10-fold dilutions of the sample and resultant slope of the curve was utilized for the calculation of the quantitative expression of both the target genes. The Ct values were recorded for both the target (GDF-9, BMP-15) and endogenous control genes (GAPDH). The data were accepted only if the NTC had no amplification.

### Interpretation of qRT-PCR

Data were quantified using the method of relative quantification in qPCR (Pfaffl 2001). The threshold cycle value of reference gene was used to normalize the target gene signals in each sample. The amount of target transcripts relative to the calibrator was calculated by subtracting the Ct value of sample reference gene from the Ct value of the sample target gene. All samples were run in duplicate and the mean value of each duplicate was used for all further calculations. The 40-ΔCt value gives the relative quantification of the transcripts.

### Statistical analysis

Statistical analysis of the data was done as per the standard procedures of Snedecor and Cochran (1994). The gene expression status of all the genes analyzed was subjected to ANOVA using a computer-aided statistical software package SPSS 16.0 for windows. All the results are expressed as the mean ± SE and statistical significance was accepted for P < 0.05.

## Results

### *In vitro* maturation of buffalo oocytes and cumulus expansion

**Figure 1:**
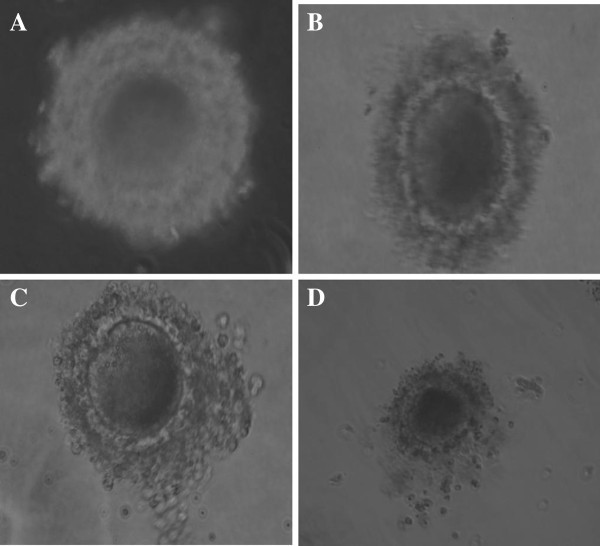
**Morphological changes of buffalo COCs during IVM.** COCs morphology observed under the microscope for: (**A**) 0 h, (**B**) 6 h, (**C**) 12 h, (**D**) 24 h. During 12 h (**C**) and 24 h (**D**) of maturation, the outermost layers of the cumulus cells become round and glistening.

Total of 692 culturable grade oocytes were collected in a batch of experiments and 20–25 oocytes were cultured in 100 μl medium at each time-point to observe the progress of IVM. The COCs morphological changes were found at different time periods of culture (Figure 1). Maturation is assessed based on cumulus expansion and polarbody extrusion. Cumulus expansion was markedly increased at 12 h after IVM cultivation and continuously increased up to 24 h. When preparing samples for expression analysis at different maturation stages, special attention was paid to selecting COCs with a uniform ooplasm and appropriate cumulus expansion to ensure an accurate status of oocytes and cumulus cells.

### Relative expression of *GDF9* and *BMP15* in buffalo oocytes during IVM

**Figure 2:**
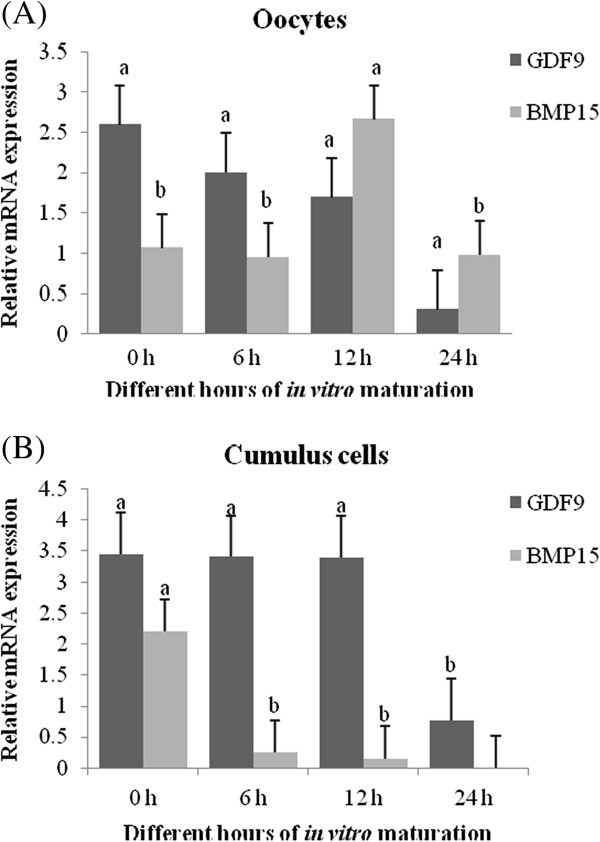
**Relative expression of*GDF9*and*BMP15*in buffalo COCs during IVM.** (**A**) Relative expression of *GDF9* and *BMP15* mRNA transcripts from oocytes. (**B**) Relative expression of *GDF9* and *BMP15* mRNA transcripts from cumulus cells. Data are presented as (mean ± SE). For each gene (*GDF9* or *BMP15*), bars with different superscripts show significant difference (P < 0.05).

The mRNA expression of *GDF9* and *BMP15* in oocytes during different hours of IVM showed differential changes (Figure 2A). Specifically, the RA of *GDF9* mRNA gradually declined throughout the time period upto 24 h of maturation and showed significant (P<0.05) variation in the RA of *GDF9* transcripts in cumulus free oocytes at between 0 h and 24 h of maturation. A significant (P<0.05) variation in the RA of *BMP15* transcript was observed in cumulus free oocytes of different maturation times. It increased between 6 h and 12 h of maturation and reduced between 12 h and 24 h.

### Relative expression of *GDF9* and *BMP15* in buffalo cumulus cells during IVM

The differential expression pattern of *GDF9* and *BMP15* mRNA transcripts in cumulus cells during different hours of IVM were presented in Figure 2B. The RA of *GDF9* remained stable upto 12 h of maturation, but decreased significantly at 24 h of maturation. However, *BMP15* showed a significant reduction in the RA between 0 h and 6 h, stayed stable upto 12 h and become undetectable at 24 h of maturation.

## Discussion

Ovarian folliculogenesis is a complex process depends upon numerous growth factors that regulate the growth and differentiation of oocytes, granulosa cells and theca cells. Oocyte-secreted *GDF9* and *BMP15* play essential roles during follicle maturation through actions on granulosa cells. *GDF9* and *BMP15* expression are less well characterized especially during IVM of COCs. Oocyte maturation is one of the important stages for the successful production of embryos *in vitro.* Moreover, it is well known that oocyte developmental potential is a reflection of proper maturation. So understanding the molecular mechanisms regulated during maturation is of great importance to optimize the IVM conditions. Since *GDF9* and *BMP15* are considered as important oocyte maturation factors during folliculogenesis (Gilchrist *et al.*^2004^), this study investigated the potential relationship between *GDF9* and *BMP15* mRNA expression from *in vitro* matured oocytes and cumulus cells during the course of *in vitro* maturation.

In the present study significant difference in the RA of *GDF9* transcripts in cumulus free oocytes, which showed gradual decline during IVM between 0h and 24 h of maturation. This is in agreement with earlier findings that suggested the *GDF9* expression to remain stable in oocyte during IVM (Prochazka *et al.*^2004^), yet showed gradual decline during oocyte maturation (Li *et al.*^2008^). Zhu *et al.* (2008) studied the temporal and spatial expression patterns of *GDF9* gene in porcine COCs throughout *in vitro* maturation and reported that *GDF9* highly transcribed in oocytes from fresh COCs, whereas expression was gradually decreased during IVM. Relative abundance of *BMP15* from denuded oocytes during different hours of IVM increased significantly (P<0.05) between 6 h and 12 h and decreased again between 12 h and 24. This increase in *BMP15* transcript may be correlated with the cumulus expansion after 12 h of maturation *in vitro*. Similar to our finding, Li *et al.* (2008) observed a specific increase in porcine *BMP15* transcript at 18 h of cumulus expansion during maturation. Zhu *et al.* (2008) reported that *BMP15* was highly transcribed in oocytes from fresh COCs and the expression gradually declined during IVM.

It was also found that *GDF9* RA in cumulus cells remained stable during IVM upto 12 h of maturation and decreased significantly between 12 and 24 h of maturation. This is in accordance with earlier studies, where RA of *GDF9* mRNA was remained stable during the first 16 h of culture and significantly reduced during later stages of culture period in cumulus cells (Prochazka *et al.*^2004^). This drastic change in the expression pattern of *GDF9* during later stages of IVM could specify any probable role of this gene in selection of dominant follicle, which warrants further investigation. Whereas, *BMP15* transcript was significantly reduced between 0 h and 6 h, stayed stable upto 12 h and undetectable at 24 h of maturation. Likewise, Zhu *et al.* (2008) reported undetectable *BMP15* expression pattern in cumulus cells during maturation. The absence of RA might be related to the degradation of transcripts after maturation of oocytes.

## Conclusion

To conclude, during maturation of COCs *in vitro*, *GDF9* mRNA showed significant reduction between 0 h and 24 h of maturation in oocytes. On the other hand, the RA of *BMP15* varied significantly (P<0.05) during IVM; It showed gradual increase between 6 h and 12 h and decreased again between 12 h and 24. However in cumulus cells, *GDF9* remained stable during IVM upto 12 h of maturation and decreased significantly between 12 h and 24 h of maturation. In case of *BMP15* significant reduction was observed between 0 h and 6 h, stayed stable upto 12 h and become undetectable at 24 h of maturation. In future, further characterization of these genes from *in vivo* matured buffalo COCs will help in better understanding of the *GDF9* and *BMP15* role in oocyte maturation of this species.

## Abbreviations

GDF9: Growth differentiation factor9
BMP15: Bone morphogenetic protein15
mRNA: Messenger Ribonucleic acid
COCs: Cumulus-oocyte complexes
IVM: *In vitro* maturation
qRT-PCR: Quantitative real-time PCR
RA: Relative abundance.
